# Single‐cell transcriptomics reveals pathogenic dysregulation of previously unrecognised chondral stem/progenitor cells in children with microtia

**DOI:** 10.1002/ctm2.702

**Published:** 2022-02-20

**Authors:** Jing Ma, Yu Zhang, Zijun Yan, Peixuan Wu, Chenlong Li, Run Yang, Xinyu Lu, Xin Chen, Aijuan He, Yaoyao Fu, Duan Ma, Weidong Tian, Tianyu Zhang

**Affiliations:** ^1^ Department of Facial Plastic and Reconstructive Surgery ENT Institute Eye & ENT Hospital Fudan University Shanghai China; ^2^ State Key Laboratory of Genetic Engineering and Collaborative Innovation Center for Genetics and Development Department of Computational Biology School of Life Sciences Fudan University Shanghai China; ^3^ School of Basic Medical Sciences Institutes of Biomedical Sciences Fudan University Shanghai China; ^4^ Children's Hospital of Fudan University Shanghai China; ^5^ Qilu Children's Hospital of Shandong University Jinan Shandong China

1

To the Editor:

Microtia, a common craniofacial birth defect worldwide, results from auricular cartilage dysplasia and is accompanied with several unfortunate physical and psychological consequences for children.^1^ However, existing treatments have adverse outcomes and its pathogenesis is poorly understood.^2^ Because characterisation of cell type composition and function in auricular cartilage can increase our understanding of microtia,^3^, ^4^ we conducted the first single‐cell transcriptomic survey in these tissues.

We collected auricular cartilage of three children with microtia and six normal controls (NC; two children, four adults), then analysed the transcriptomes of 47 214 total cells after stringent quality control (Figure 1A and Figure S1A–F; Table S1). We detected seven cell types through unsupervised clustering analysis combined with previous reports of cell type‐specific marker genes (Figure 1B and Figure S1G). The four cartilage‐related cell types included quiescent chondral stem/progenitor cells (CSPCs; expressing *EGR1* ^5^ *HES1*,^6^ *COL2A1* and *CYTL1*), which could directly differentiate into chondrocytes (Chonds; *COL2A1* and *CYTL1*); quiescent stromal stem/progenitor cells (SSPCs; *EGR1*, *HES1*, *COL1A1* and *LUM*) and stromal cells (SCs; *COL1A1* and *LUM*), whereas other cell types included immune cells, perivascular cells and endothelial cells (Figure 1C,D and Figures S1H,J and S2; Table S2). Gene ontology (GO) terms enriched in the above cell types also showed their specific functions (Figure 1D).

**FIGURE 1:**
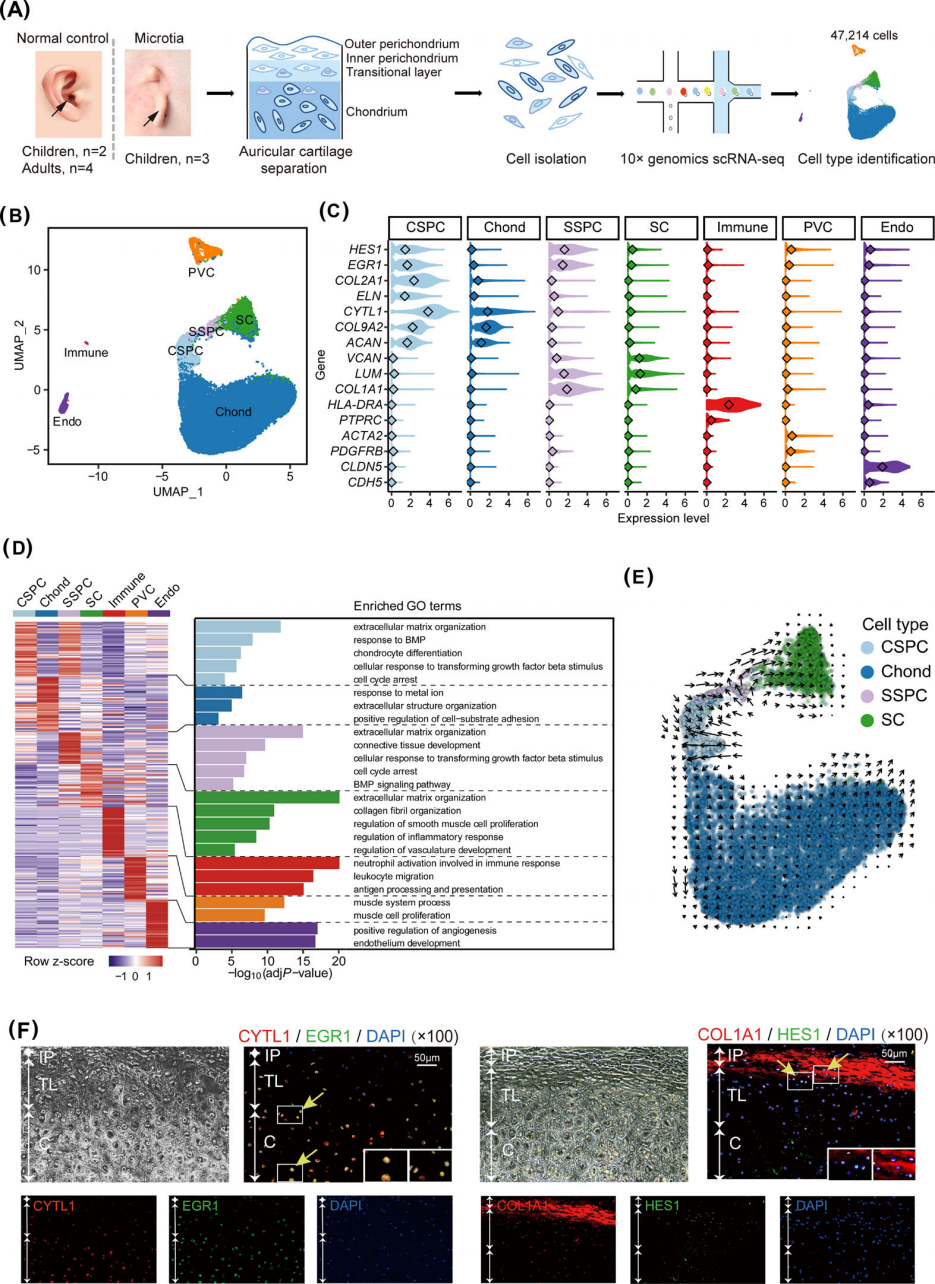
Seven cell types with transcriptional signatures determined by scRNA‐seq analysis of human auricular cartilage. (A) The flowchart of single‐cell transcriptome analysis on human auricular cartilage. Black arrows indicate the parts of auricular cartilage used for single‐cell isolation. (B) UMAP projection of 47 214 total cells. Each dot represents a cell. (C) Violin plots displaying the normalised expression levels of selected marker genes for each cell type. The diamond within each violin represents the median value. (D) DEGs among all the cell types. Left: Heatmap showing row‐scaled average gene expression levels of top 50 specifically expressed genes in each cell type. Each column corresponds one cell type and each row corresponds one gene. Right: Bar plots displaying the representative GO terms enriched in each cell type. (E) Developmental trajectory of CSPCs, Chonds, SSPCs and SCs (43 948 cells) inferred by RNA velocity and visualised on the UMAP projection. Arrows indicate possible directions of development. (F) The location of CSPCs and SSPCs in human auricular cartilage tissue. White arrows indicate the layering of human auricular cartilage, yellow arrows with small boxes point the specific location of the cells. The image in the large box on the lower right is an enlargement of the smaller box. DAPI with blue indicates the nucleus. Left: EGR1 (green) and CYTL1 (red) double‐positive CSPCs exist in the transitional layer and chondrium. Right: HES1 (green) and COL1A1 (red) double‐positive SSPCs exist in the transitional layer and inner perichondrium. Scale bar: 50 μm. scRNA‐seq, single‐cell RNA sequencing; CSPC, chondral stem/progenitor cell; Chond, chondrocyte; SSPC, stromal stem/progenitor cell; SC, stromal cell; Endo, endothelial cell; Immune, immune cell; PVC, perivascular cell; GO, gene ontology; IP, inner perichondrium; TL, transitional layer; C, chondrium

Pseudotime trajectory analysis revealed two distinct developmental lineages: CSPCs differentiation into Chonds (chondral lineage), and SSPCs differentiation into SCs (stromal lineage) (Figure 1E). Moreover, immunofluorescence showed that SSPCs were specifically found in the transitional layer and inner perichondrium, whereas CSPCs were specifically located in the chondrium and transitional layer, which differed from published descriptions of ‘cartilage stem cells’ (located in the outer perichondrium; similar to mesenchymal stem cells [MSCs] that specifically co‐express CD44 and CD90 and also exhibit the capacity for differentiation into chondrocytes, osteocytes and adipocytes in vitro)^7^ (Figure 1F and Figure S1I).

Subclustering analysis showed four subtypes in the chondral lineage, with CSPCs progressing to early chondrocytes (ECs), to intermediate chondrocytes (ICs) and finally to late chondrocytes (LCs) (Figure 2A,B and Figure S3A,B; Table S3). Stemness markers, chondrocyte differentiation and extracellular matrix (ECM)‐related genes were highly expressed in CSPCs, but were gradually downregulated from ECs to LCs, whereas cartilage degeneration‐related genes were progressively upregulated from CSPCs to LCs (Figure 2C and Figure S3C). GO analysis supported the above findings (Figures 1D and 2D). Three subtypes were identified in the stromal lineage (Figure 2E and Figure S3D,E; Table S3), with SSPCs differentiating into SC1 or SC2 in parallel (Figure 2F). GO analysis suggested that SC1 could participate in communication with other cell types outside the cartilage, whereas SC2 likely functioned in cartilage formation (Figure 2G,H).

**FIGURE 2:**
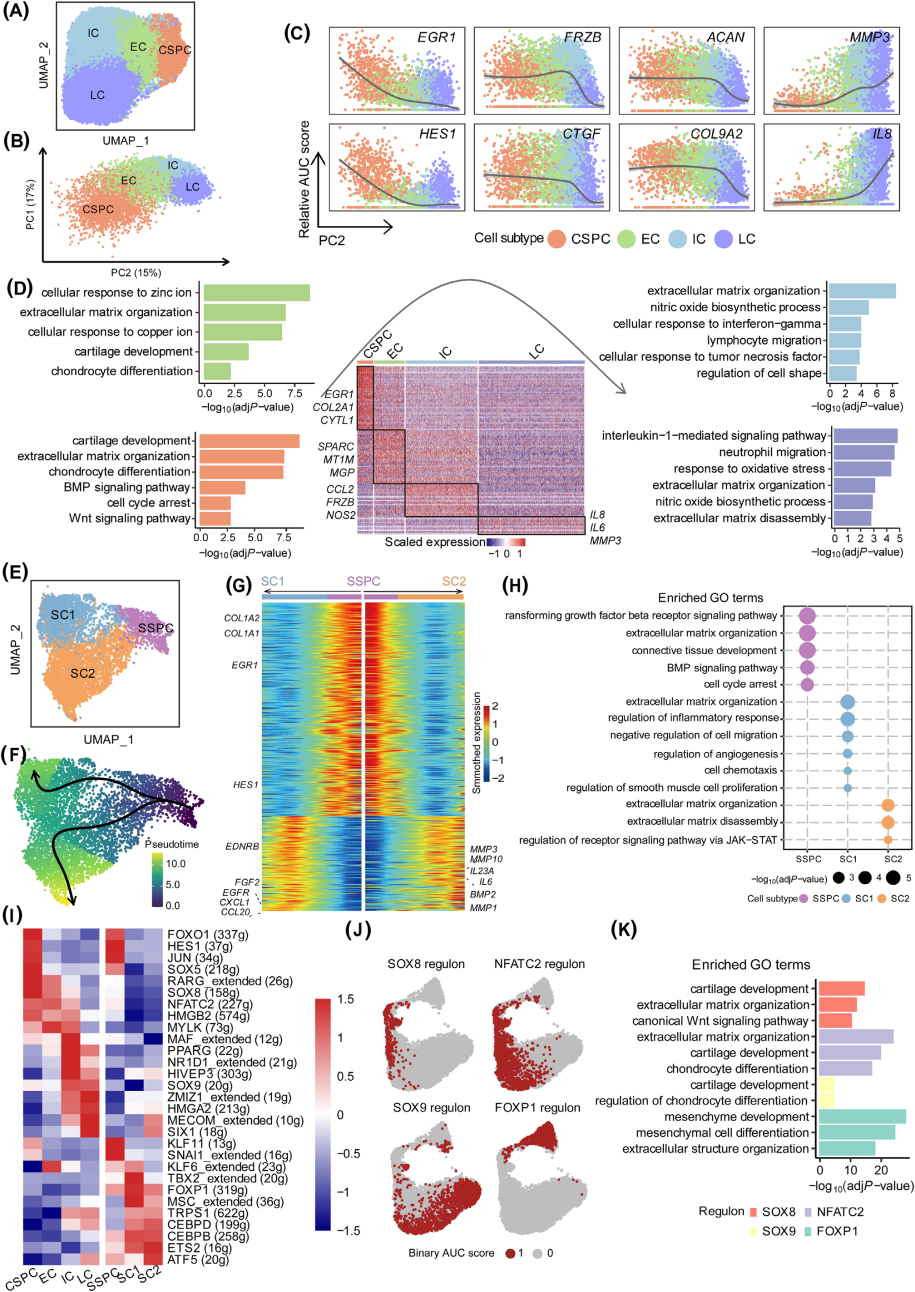
The subtypes of the chondral and stromal lineages based on the developmental trajectory. (A) UMAP projection of 38 246 cells in the chondral lineage. (B) PCA plot showing the developmental trajectory of four subtypes in the chondral lineage based on gene expression patterns along PC2. (C) Relative gene expression patterns of representative genes essential for the chondral lineage along the PC2 dimension. (D) Heatmap showing gene expression levels of top 50 subtype‐specific genes in four subtypes of the chondral lineage. Representative GO terms enriched in four cell subtypes are shown in turn clockwise. (E) UMAP projection of 5702 cells in the stromal lineage. (F) Developmental trajectory of the stromal lineage inferred by Slingshot and visualised on the UMAP by pseudotime score. The pseudotime score from dark blue to light yellow indicates state from early to terminal. (G) Heatmap of smoothed gene expression of subtype‐specific genes from SSPC to SC1, and SSPCs to SC2, respectively. Smoothed gene expression from dark blue to dark red represents the expression from low to high. The representative genes are labelled on both sides of the graph. (H) Dot plots displaying the representative GO terms enriched in the stromal lineage. (I) The regulons enriched in the chondral and stromal lineage. Heatmap showing the scaled AUC score of the enriched regulons. The number of predicted target genes for each regulon is shown in the parenthesis. The AUC score from dark blue to dark red indicates the regulon activity from low to high. (J) UMAP plots of binary activities of representative regulons SOX8, NFATC2, SOX9 and FOXP1. Red dots represent regulon activated in this cell. (K) Bar plots displaying the representative GO terms enriched in SOX8, NFATC2, SOX9 and FOXP1 regulons. EC, early chondrocyte; IC, intermediate chondrocyte; LC, late chondrocyte; g, genes

Single‐cell regulatory network inference and clustering^8^ identified four major regulons, that is, transcription factors (TFs) and their targets, included SOX8 regulon for CSPCs, NFATC2 regulon for ECs, SOX9 regulon for ICs and LCs and FOXP1 regulon for the stromal lineage, which enriched for cartilage development‐related GO terms (Figure 2I–K and Figure S4; Table S4).

Focusing on the chondral lineage, we found a higher proportion of CSPCs and ECs, but fewer ICs and LCs, in children compared with adults, among NC group (Figure 3A). Further, approximately half of the differentially expressed genes (DEGs) were shared among at least two subtypes, whereas relatively few DEGs were specific to a single subtype (Figure 3B and Figure S5A,B; Table S5). Upregulated and downregulated DEGs enriched for ageing and ECM formation‐related terms, respectively, in most or all subtypes of adults (Figure 3C and Figure S5C,D).

**FIGURE 3:**
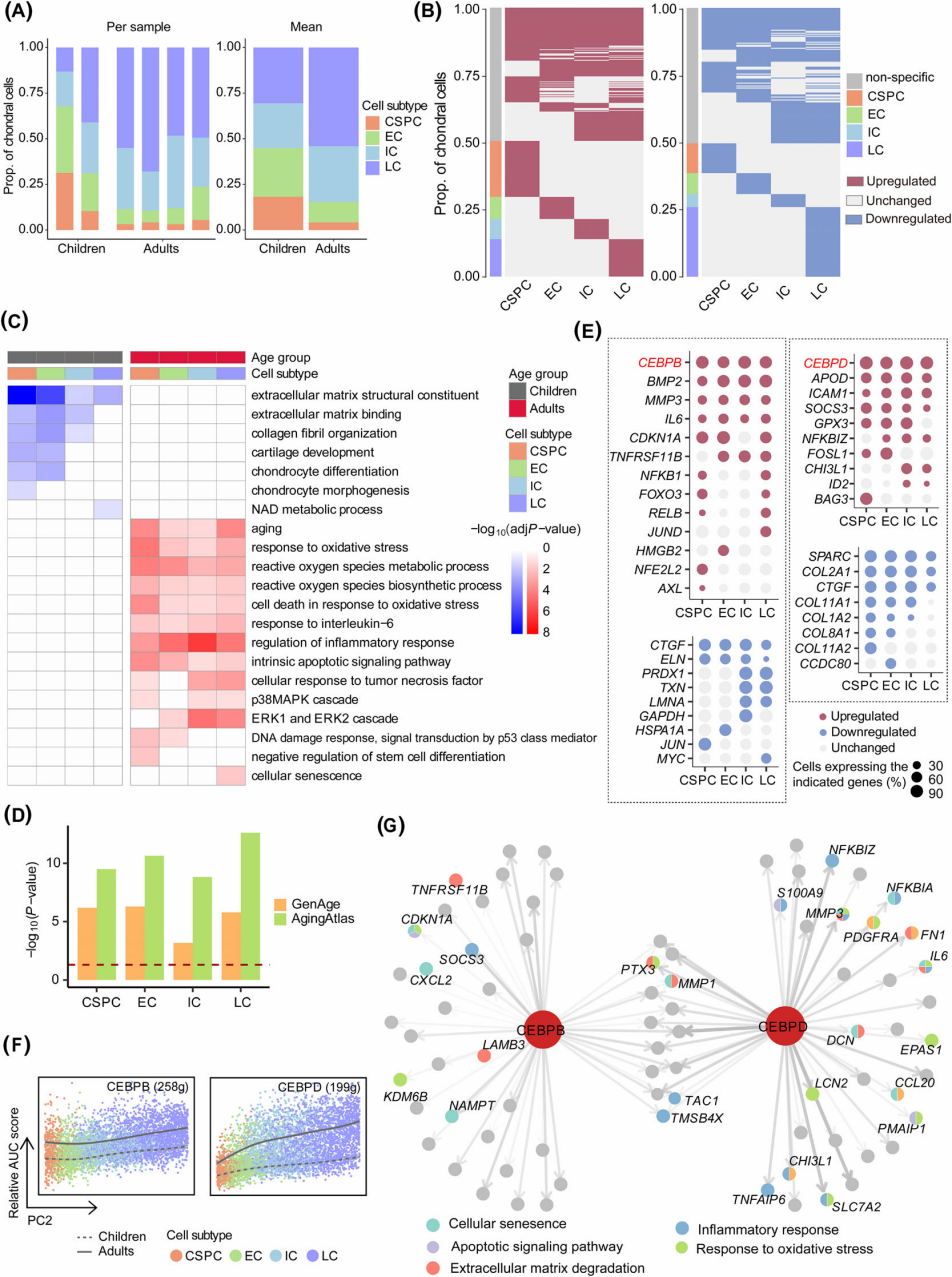
Integrative analysis of the effect of age on the chondral lineage. (A) Stacked bar charts showing relative proportions of four subtypes in the chondral lineage of children and adults from NC group. (B) Heatmaps showing the distribution of DEGs between children and adults from NC group in each subtype of the chondral lineage. Stacked bar charts on the left of the heatmaps show the relative proportions of DEGs in each subtype; gray blocks in the heatmaps denote DEGs shared by at least two subtypes and the others are subtype‐specific DEGs. (C) Representative GO terms enriched in each subtype of children and adults from NC group. (D) Bar plots showing the enrichment degree between DEGs with GenAge (orange bar) and Aging Atlas (green bar) databases. Red dashed line represents the significant cutoff. (E) Dot plots showing expression patterns of upregulated (up) and downregulated (down) DEGs of four subtypes included (left) or not included (right) in the Aging Atlas or GenAge database. Dot size represents the proportion of cells expressing specific gene in the subtype. (F) AUC score of CEBPB and CEBPD regulons along the PC2 dimension. Solid line represents adults, whereas dashed line represents children. The number of predicted target genes for each regulon is shown in the parenthesis. g, genes. (G) The GRN of CEBPB and CEBPD in the chondral lineage. Line width indicates the level of GENIE3 weights. Upregulated TF nodes are coloured in red. Gene nodes are coloured according its category of enriched GO terms

Additionally, we found that DEGs in each subtype were significantly enriched in the Aging Atlas^9^ and GenAge^10^ databases (Figure 3D), in which the TF, CEBPB, was upregulated in all subtypes in the chondral lineage of adults (Figure 3E). CEBPD, the interaction partner of CEBPB, was also upregulated in each subtype (Figures 3E and Figure S5E). They exhibited the highest co‐expression/regulatory activity (Figure S5F), and their regulatory activities were upregulated in all subtypes of adults (Figure 3F). Further gene regulatory network (GRN) analysis confirmed that upregulated DEGs targeted by CEBPB and CEBPD were significantly enriched in ageing‐related terms (Figure 3G and Figure S5G,H).

When exploring the role of the chondral lineage in microtia, a lower proportion of CSPCs was found in microtia compared with NC children (Figure 4A). Microtia‐associated DEGs (MADEGs) were obtained after excluding the influence of gender factor, and CSPCs had the highest percentage of differentially expressed marker genes among all subtypes (Figure S6A–C; Table S6). In particular, CSPCs showed more significant enrichment than other subtypes for ‘ECM structural constituents’, ‘cartilage development’, ‘cellular respiration’ among downregulated MADEGs, and ‘response to oxidative stress’, ‘apoptotic signaling pathway’ among upregulated MADEGs (Figure 4B,C). Time course analysis identified four gene groups potentially influencing the chondral lineage trajectory of microtia (Figure 4D and Figure S6D,E; Table S7). The TF group, predominantly comprised TFs, was responsible for transcriptional regulation (Figure 4D). In this group, genes showed the highest transcription levels in CSPCs, and differences in gene expression were largest in CSPCs differentiating into ECs between microtia and NC children (Figure 4D).

**FIGURE 4:**
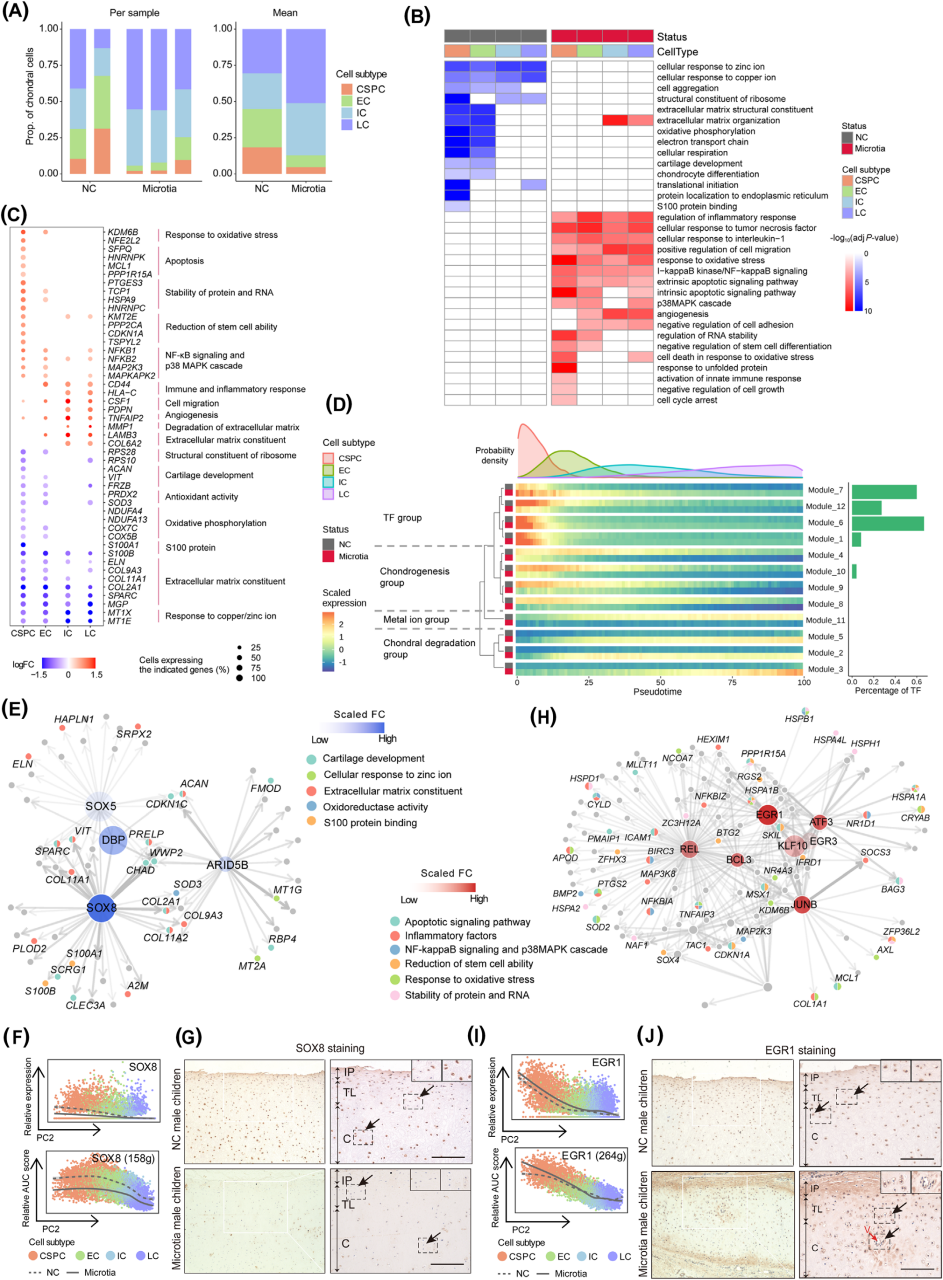
Aberrant gene expression profiles in cell subtype‐specific manners in microtia. (A) Stacked bar charts showing relative proportions of four subtypes in the chondral lineage of microtia and NC children. (B) Representative GO terms enriched in each subtype of microtia and NC children. (C) Dot plot showing the representative MADEGs in each subtype between microtia and NC children from GO analysis. (D) Heatmaps showing gene expression changes along with pseudotime between microtia and NC children in each subtype. The probability density plot of four subtypes is above the heatmap; bar plot showing the percentage of TF in each module is on the right; hierarchical clustering tree of modules and biological functions of each module group is on the left. (E) The downregulated GRN in CSPCs of microtia. Nodes transparency represents scaled fold change (FC) of TFs; node size represents the page rank scores of TFs; line width represents the Genie3Weights. (F) Relative gene expression and AUC score of SOX8 regulons along the PC2 dimension. Solid line represents microtia, whereas dashed line represents NC children. The number of predicted target genes for regulon is shown in the parenthesis. g, genes. (G) IHC staining of SOX8 in chondrium and transitional layer of auricular cartilage from normal male children (up panels, *N* = 3) and male children with microtia (down panels, *N* = 3). Brown staining showed HRP staining. Nuclei counterstained by haematoxylin. In each panel: left, white rectangles indicate enlarged areas shown in the right; right, the inset shows the enlarged area indicated by the black dashed rectangle; the black arrows illustrate cells. (H) The upregulated GRN in CSPCs of microtia. Node transparency represents scaled FC of TFs; node size represents the page rank scores of TFs; line width represents the Genie3Weights. (I) Relative gene expression and AUC score of EGR1 regulons along the PC2 dimension. Solid line represents microtia, whereas dashed line represents NC children. (J) IHC staining of EGR1 in chondrium and transitional layer of auricular cartilage from normal male children (up panels, *N* = 3) and male children with microtia (down panels, *N* = 3). In each panel: left, white rectangles indicate enlarged areas shown in the right; right, the inset shows the enlarged area indicated by the black dashed rectangle; the red arrows show vessels in the chondrium; the black arrows illustrate cells. IP, inner perichondrium; TL, transitional layer; C, chondrium; V, vessel. Scale bar: 200 μm

To determine which TFs could contribute essential pathogenic functions to microtia development, we used the PageRank algorithm to assess this potential for TFs in GRNs constructed with downregulated or upregulated MADEGs of CSPCs in microtia, respectively (Figure 4E,H). In downregulated GRN, the CSPC‐specific SOX8 TF (Figure 2J) had the highest PageRank score and greatest decrease in expression (Figure 4E,F and Figure S7), targeting MADEGs involved in ECM organisation and cartilage development. In upregulated GRN, quiescent stem cell marker EGR1 showed the higher differential expression and PageRank score simultaneously, primarily targeting MADEGs related to the response to oxidative stress (Figure 4H,I and Figure S8). Immunohistochemistry verified that SOX8 decreased, while EGR1 increased, in protein expression in CSPCs of microtia (Figure 4G,J).

Here, we systematically annotate cartilage‐related cell subtypes in human auricular cartilage by single‐cell RNA sequencing. This study also represents the first description of CSPCs in the chondrium, potentially enabling directed chondrogenic differentiation, and shows that dysregulation of cartilage development by SOX8, and dysregulation of the response to oxidative stress by EGR1, in CSPCs may be causative mechanisms driving microtia. While future work will explore these possibilities, these findings build a theoretical foundation for advanced exploration of the microtia pathogenesis and offer new avenues for potential treatments.

## Supporting information

Supporting Information

Supporting Information

Supporting Information

Supporting Information

Supporting Information

Supporting Information

Supporting Information

Supporting Information
