# Supplementary material for: Single‐cell transcriptomics reveals pathogenic dysregulation of previously unrecognised chondral stem/progenitor cells in children with microtia
Source: Clin Transl Med. 2022 Feb 20;12(2):e702. doi: 10.1002/ctm2.702 (PMC8858629; doi:10.1002/ctm2.702)
Supplement: Supplementary file 1 — Supporting Information [file CTM2-12-e702-s003.docx]

**Single Cell Transcriptomics Reveals Pathogenic Dysregulation of Previously Unrecognized Chondral Stem/Progenitor Cells in Children with Microtia**

Jing Ma^1#^, Yu Zhang^2#^, Zijun Yan^2#^, Peixuan Wu^3^, Chenlong Li^1^, Run Yang^1^, Xinyu Lu^1^, Xin Chen^1^, Aijuan He^1^, Yaoyao Fu^1^, Duan Ma^3,4*^, Weidong Tian^2,4,5*^, Tianyu Zhang^1*^

1 Department of Facial Plastic and Reconstructive Surgery, ENT institute, Eye & ENT Hospital, Fudan University, Shanghai 200031, China;

2 State Key Laboratory of Genetic Engineering and Collaborative Innovation Center for Genetics and Development, Department of Computational Biology, School of Life Sciences, Fudan University, Shanghai 200436, China;

3 School of Basic Medical Sciences, Institutes of Biomedical Sciences, Fudan University, Shanghai 200032, China;

4 Children’s Hospital of Fudan University, Shanghai 201102, China;

5 Qilu Children’s Hospital of Shandong University, Jinan 250022, Shandong, China

* Corresponding author

# These authors contributed equally to this work and shared first authorship.

**Methods and Materials**

**Patients and sample collection**

This study was reviewed and approved by the Institutional Research Ethics Committee of Eye & ENT Hospital of Fudan University (2020069). The cartilage samples were from the residual auricle of three children with third-degree isolated microtia (8-10 years old) and concha of auricle of six NCs (two children, 7-8 years old and four adults, 20-60 years old) (Table S1). The third-degree microtia is the most common case for surgery. NC samples were the auricular cartilage tissues from patients with tympanitis. All of them had no extra physical abnormalities. The information of all samples was described in Table S1. After the perichondrium was stripped on ice immediately, auricular cartilage tissue was collected and stored in ice-cold phosphate buffer saline (PBS) for the preparation of cell suspension.

**Tissue dissociation and single cell isolation**

All samples were minced into ~1 mm^2^ pieces and digested for 10 minutes with trypsin at 37°C to remove residual tissue attached to cartilage tissue. Then, the tissues were digested in 0.2% Collagenase, Type Ⅱ (Thermo Fisher Scientific, NV, USA) with shaking at 37°C for 2 hours and collected at 30 minutes intervals until 14 hours. After stopping digesting by adding Dulbecco's modified Eagle's medium: F12 (Gibco, NM, USA) containing 10% fetal bovine serum (FBS) (Gibco), the dissociated cells were filtered through a 30 µm cell strainer (BD Falcon, NJ, USA) and centrifuged at 300 g for 5 minutes at 4°C. The single cell suspension in 0.04% bovine serum albumin (BSA) (Gibco) in PBS was used for scRNA-seq platform (10x Genomics, USA).

**Single-cell library preparation and sequencing**

The cell suspension was loaded onto the Chromium single-cell controller (10x Genomics, USA) to generate single-cell gel beads in the emulsion (GEMs) by using Single Cell 3′ Library and Gel Bead Kit V3 (10x Genomics, USA) and Chromium Single Cell A Chip Kit (10x Genomics, USA) (performed by CapitalBio Technology, Beijing). In short, approximately 10,000 cells were added to each channel, and about 6,000 cells were recovered. The captured cells were lysed, and the released RNA was barcoded through reverse transcription in individual GEMs. Reverse transcription was performed at 53°C for 45 minutes, followed by 85°C for 5 minutes, and hold at 4°C on a S1000TM Touch Thermal Cycler (Bio Rad, USA). The cDNA was generated and then amplified for the construction of scRNA-seq libraries and finally sequenced on Novaseq6000 (Illumina, USA).

**ScRNA-seq data processing**

Digital gene expression matrices were generated for each sample using the Cell Ranger (version 3.1.0) pipeline coupled with human reference version hg19, then were analyzed by R software (version 4.0.0) with the Seurat (51) package (version 3.1.5). In brief, after removal of low-quality cells meeting one of the following criterias: (i) <2000 unique molecular identifiers (UMIs), or (ii) >6000 or <800 genes, or (iii) >10% UMIs derived from the mitochondrial genome, the gene expression matrices were normalized by the NormalizeData function, and the top 4000 highly variable genes (HVGs) were selected using the FindVariableGenes function. To reduce the dimensionality of the datasets, the RunPCA function was conducted on linear transformation scaled data generated by the ScaleData function. The RunHarmony function of harmony^1^ package (version 1.0) was used to eliminate batch effects from different samples in the principal component (PC) space with default parameters. The top 15 harmony components were processed using the RunUMAP function to embed and visualize the cells in a two-dimensional map. Cells were clustered utilizing the FindNeighbors and FindClusters functions with the parameter resolution as 1.2 based on the first 50 harmony components.

**Cell type annotation in human auricular cartilage**

To annotate cell clusters, DEGs of each cluster were identified by performing FindAllMarkers function with default parameters. Only genes expressed in at least 10% cells of one tested cluster, with fold change (FC) >1.5 and Bonferroni-corrected p value < 0.05 were regarded as DEGs. The cell clusters were then annotated according to the DEGs combined with the curated known cell markers from literature. Clusters expressing the same cell markers were merged.

The enriched biological functions of DEGs in each cell type were analyzed by enrichGO function of clusterProfiler^2^ package (version 3.16.0) in R. The enriched GO terms were filtered with the p value adjusted by Benjamini-Hochberg (BH) method (adjP-value) <0.05.

**Trajectory analysis using RNA velocity**

Development trajectory analysis on cartilage-related cells was performed using RNA velocity (<http://velocyto.org/>) as instructed^3^. Briefly, the spliced and un-spliced reads for each gene were firstly quantified by the velocyto python package (version 0.17.11) with human genome reference hg19. Then the output loom file was used to calculate velocity of each gene following the pipeline of Velocyto.R (version 0.6). Finally, the visualization of RNA velocity map was projected onto the UMAP plot. The 10,000 cells were sampled for this analysis.

**Identifying transcriptionally distinct subtypes in chondral and stromal lineage**

The data of the chondral and stromal lineage were extracted from the integrated dataset firstly. Next, normalization, scaling, principal component analysis (PCA), and clustering were performed as described above. The difference was, the top 3000 HVGs were selected, the cells were re-clustered with the first 80 harmony components and resolution as 0.3 for chondral lineage, and the first 50 harmony components and resolution as 0.15 for stromal lineage. DEGs of each subtype were obtained and analyzed as described above. Cells in chondral lineage were aligned on a linear trajectory along with PC2 corrected by harmony, and cells in stromal lineage were aligned on a pseudo-time trajectory using Slingshot package (version 1.6.1) with default parameters (<https://github.com/kstreet13/slingshot>).

**GRN analysis of the chondral and stromal lineage**

The GRN and regulon activities in chondral and stromal lineage were characterized by single-cell regulatory network inference and clustering (SCENIC) package (version 1.1.3) (<https://github.com/aertslab/SCENIC>). In details, the expression matrix was loaded onto GENIE3 package (version 1.10.0) for building the initial GRN between TFs and putative target genes. Indirect targets in the regulon data were pruned by TF motifs, which were obtained using the RcisTarget package (version 1.8.0) with hg19-tss-centered-10kb-7species.mc9nr.feather, hg19-500bp-upstream-7species.mc9nr.feather. The regulon activity in each cell was measured with area under the recovery curve (AUC) by the AUCell package (version 1.10.0). Differential activated regulon (DARs) were obtained by t-test implemented in limma package (version 3.44.1) with adjP value < 0.05 and FC >2.

The representative regulon of a subtype was defined as following two items: the regulon activity was the most specific, and the expression levels of TF in the regulon were also the most specific in the subtype. The specificity of regulon activity was measured by the difference between the highest and the second highest mean AUC score, and the specificity of TF expression was measured by the difference between the highest and the second highest mean expression level in all subtypes. GO enrichment of genes in DARs was the same as DEGs analysis descried above.

**Identification of DEGs between children and adults in NC group**

DEGs between children and adults in each cell subtype of the chondral lineage were identified and analyzed as before.

**Aging-associated transcriptional variation analysis**

To observe the effects of age on different cell subtypes in chondral lineage, the age-relevant coefficient of variation (CV) analysis was performed as described in previous studies^4^.

**Gene set Analysis**

The module scores for gene expression programs in single cell were calculated by the AddModuleScore function in Seurat. Gene sets were obtained from the MSigDB database (<https://www.gsea-msigdb.org/gsea/msigdb/>). One-tail wilcox test was used to compare differences between children and adults. Gene sets related to age were obtained from the GenAge database (<https://genomics.senescence.info/genes/index.html>) and the Aging atlas database (<https://bigd.big.ac.cn/aging/index>), respectively. R function fisher.test was used to evaluate the significance of the association between DEGs and gene sets in GenAge or Aging atlas, respectively. A p value < 0.05 was considered statistically significant.

**Construction of GRN of CEBPB and CEBPD**

The expression correlation of CEBPB with other TFs were calculated by Pearson correlation coefficient using cor.test function, as well as for the regulatory activity correlation analysis of CEBPB regulon with other regulons. The GRN of CEBPB, CEBPD and predicted target genes was visualized by R package ggraph (version 2.0.2). In GRN, correlations of TFs and target genes were represented by Genie3Weights from GENIE3, and importance of TFs was measured by PageRank algorithm using igraph package (version 1.2.5).

**Identification of microtia-associated DEGs and critical gene modules, and construction of GRN in microtia**

Differential expression analysis was performed in each cell subtype of the chondral lineage as before. To exclude the impact of gender factor, firstly, we identified gender-associated genes (GAGs) between the normal adult male group (sample C2) and the normal adult female group (sample C1 and C3). Next, the DEGs between NC and microtia children were identified, and the DEGs overlapped with GAGs were regarded as gender-associated DEGs (GADEGs). Then, the microtia-associated DEGs (MADEGs) were obtained by excluding GADEGs from DEGs between NC and microtia children. The GO analysis for MADEGs was also performed as before.

Time course analysis of the chondral lineage was executed by maSigPro^5^ package (version 1.60.0). In brief, firstly, the union gene set was from MADEGs with normalized expression levels more than 0.25 in each cell subtype. Secondly, all cells in chondral lineage were ranked by PC2, divided into 100 intervals equally, and assigned time labels as input of maSigPro. Then, maSigPro with a two steps regression strategy was carried to find critical genes with not only significant temporal expression changes but also significant trend differences between NC and microtia. Thirdly, the critical genes were clustered to modules and groups by hierarchical clustering. GO enrichment of genes in each module was performed as before. TF proportion of each module was calculated with help of Human Transcription Factors (HTF) database.

The GRN of differentially up/down-regulated TF and predicted target genes in CSPCs of microtia was visualized as before.

**Ligand-receptor interaction analysis of the chondral lineage**

The CellPhoneDB ^6^ software (<https://github.com/Teichlab/cellphonedb>, version 2.1.2) was used to identify ligand–receptor pairs in NC and microtia samples, respectively. In short, the mean of the average receptor expression level of a subtype and the average ligand expression level of the interacting subtype were calculated by randomly permuting the subtype labels of all cells 1,000 times. The pairwise comparisons were performed between all subtypes and a likelihood of p value was used for filtering out the false-positive interaction. Interactions with p value < 0.05 were considered to be real.

**Immunofluorescence (IF) staining**

Tissues were fixed in 4% paraformaldehyde (PFA) and dehydrated, then embedded in paraffin and cut into sections with a thickness of 4 μm. Sections were deparaffinized in a series of 100% xylene washes (Sinopharm, China), rehydrated in graduated alcohol (100%, 100%, 100%, 95%, and 80%), permeabilized with 0.3% Triton X-100 for 20 minutes at 37°C, and heated for antigen retrieval. After 30-minute 3% BSA (Solarbio, China) blocking step at room temperature, the slides were incubated with primary antibodies HES1 (mouse, 1:50; ab119776, England, Abcam), EGR1 (mouse, 1:100; MB0443, China, Abmart), COL1A1 (rabbit, 1:100; 72026T, Cell signaling technology, MA, USA), and CYTL1 (rabbit, 1:100; 15856-1-AP, Proteintech, IL, USA) overnight at 4°C. Following washed with PBS twice, the slides were subjected to the fluorophore conjugated secondary antibodies: goat anti rabbit RedX (1:400; 111-295-003, Jackson, PA, USA), goat anti mouse FITC (1:400; 115-095-003, Jackson) for 45 minutes at 37°C. Then slides counterstained and mounted with DAPI Fluoromount-G (1:400; 0100-20, SouthernBiotech, AL, USA) for 10 minutes in the dark. Finally, the slides were rinsed in distilled water, dehydrated, mounted and imaged with inverted fluorescence microscope (Olympus, Japan). The images were merged, and brightness was adjusted using ImageJ software (Version 1.53a, <https://imagej.nih.gov/ij/>).

**Immunohistochemical (IHC) staining**

The tissues were fixed, embedded, sectioned, deparaffinized, rehydrated, permeabilized and antigen retrieved according to the procedure above. The 3% H_2_O_2_ was applied to block endogenous peroxidase activity before a 30-minute 3% BSA blocking step at 37°C, and then the slides were incubated with primary antibodies SOX8 (rabbit, 1:100; 20627-1-AP, Proteintech) and EGR1 (rabbit, 1:100; 22008-1-AP, Proteintech) respectively. After incubation overnight at 4°C, the sections were washed with PBS and subjected to the horseradish peroxidase (HRP) conjugated secondary antibody (1:500; 111-035-045, Jackson), following visualized with diaminobenzidine (DAB) staining (VECTOR, CA, USA). Counterstaining was then performed with hematoxylin. After cover-slipped, slides were imaged using EVOS^TM^ Microscope M5000 Imaging System (Invitrogen, CA, USA). The brightness of images was adjusted using ImageJ software.

**Masson’s Trichrome Staining**

After deparaffinized and rehydrated, the slides were immersed in Bouin’s solution at 56°C for 1 hour. Then the slides were stained in Weigert’s iron hematoxylin working solution for 10 minutes and in Biebrich scarlet-acid fuchsin for 10 minutes, successively. Next, the slides were subjected to the phosphotungstic-phosphomolybdic acid solution for incubation and aniline blue for dyeing, following by the differentiation in 1% acetic acid solution. Mounted slides were imaged using EVOS^TM^ Microscope M5000 Imaging System. The brightness of images was adjusted using ImageJ software.

**Ethics approval and consent to participate**

This study was reviewed and approved by the Institutional Research Ethics Committee of Eye & ENT Hospital of Fudan University (2020069).

**Consent for publication**

This manuscript has been read and approved by all authors. Each author agrees to accept responsibility for its contents. All authors warrant that the article is original, is not submitted or under consideration by another journal, has not been previously published, and does not infringe upon any copyright or other proprietary right of any third party.

**Data availability statement**

Raw sequencing data and processed data are available through the NCBI Gene Expression Omnibus (GEO) under accession number GSE179135. All other data supporting the findings of this study are available within the article and its Supplementary Information files or from the corresponding authors upon reasonable request. Special code will be available upon reasonable request.

**Conflict of interest**

The authors declare no conflict of interest.

**Funding**

This project was funded by National Natural Science Foundation of China (81771014, 81800920, 31871325, 32170667); Natural Science Foundation Project of Shanghai Science and Technology Innovation Action Plan (20ZR1409900).

**Authors’ contributions**

Z.T.Y., T.W.D., M.D. and M.J. conceived and supervised this project. L.C.L., Y.R., L.X.Y., C.X., H.A.J. and F.Y.Y. coordinated sample collection and clinical data interpretation. Z.Y. and Y.Z.J. performed bioinformatics analysis. W.P.X. performed immunostaining analysis. M.J., Z.Y., Y.Z.J., and W.P.X. wrote the manuscript.

**Supplementary References**

1. Korsunsky I, Millard N, Fan J, et al. Fast, sensitive and accurate integration of single-cell data with Harmony. *Nat Methods.* 2019;16(12):1289-1296.

2. Yu GC, Wang LG, Han YY, He QY. clusterProfiler: an R Package for Comparing Biological Themes Among Gene Clusters. *Omics.* 2012;16(5):284-287.

3. La Manno G, Soldatov R, Zeisel A, et al. RNA velocity of single cells. *Nature.* 2018;560(7719):494-498.

4. Wang S, Zheng Y, Li J, et al. Single-Cell Transcriptomic Atlas of Primate Ovarian Aging. *Cell.* 2020;180(3):585-600 e519.

5. Nueda MJ, Tarazona S, Conesa A. Next maSigPro: updating maSigPro bioconductor package for RNA-seq time series. *Bioinformatics.* 2014;30(18):2598-2602.

6. Efremova M, Vento-Tormo M, Teichmann SA, Vento-Tormo R. CellPhoneDB: inferring cell-cell communication from combined expression of multi-subunit ligand-receptor complexes. *Nat Protoc.* 2020;15(4):1484-1506.

**Supplementary Figure Legend**

**
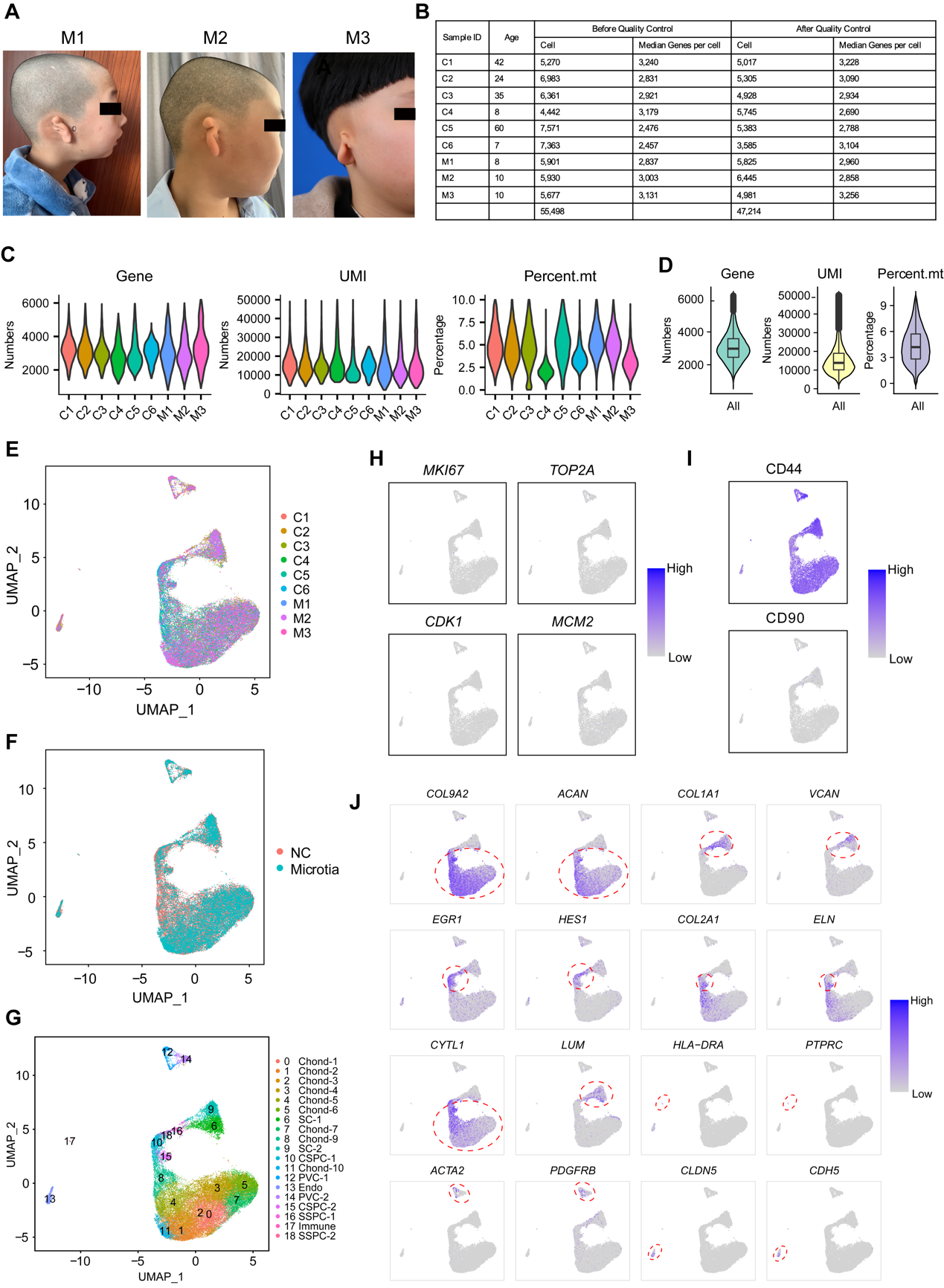
**

**Supplementary Figure 1. Primary data analysis and distribution of clusters across different samples.**

(A) Pictures of three microtia children.

(B) Summary of age of samples, the number of cells, median genes per cell before and after quality control from microtia (M) and NC (C). After strict quality control, the transcriptomes were obtained from a total of 47,214 single cells (17,251 cells for microtia and 29,963 cells for NC) for further analysis.

(C-D) Violin plots showing the number of genes (left), UMI (middle) and percentage of mitochondrial genes (right) captured in each sample (C) and all samples (D) after filtering. After strict quality control, expression of a median of 2,978 genes, 13,838 unique molecular identifiers (UMI), and less than 10% of the mitochondrial genes per cell were detected. In (D), the horizontal line within each box represents the median, and the top and bottom of each box indicate the 75th and 25th percentile.

(E-G) The UMAP projection of cells in human auricular cartilage from all samples integrated by harmony, colored by batch (E), status (F) and cluster (G).

(H) UMAP plot of the normalized expression of *MKI67*, *TOP2A*, *CDK1*, and *MCM2*, representative genes indicating cells in proliferative status in all cells.

(I) UMAP plot of the normalized expression of CD44 and CD90 in all cells. Descriptions in past studies of “cartilage stem cells” in the outer perichondrium expressed these two markers. This early description was more similar to mesenchymal stem cells (MSCs) that specifically express CD44 and CD90 and also exhibited the capacity for differentiation into chondrocytes, osteocytes, and adipocytes *in vitro*.

(J) UMAP plot of the normalized expression of selected marker genes used for cell types identification.


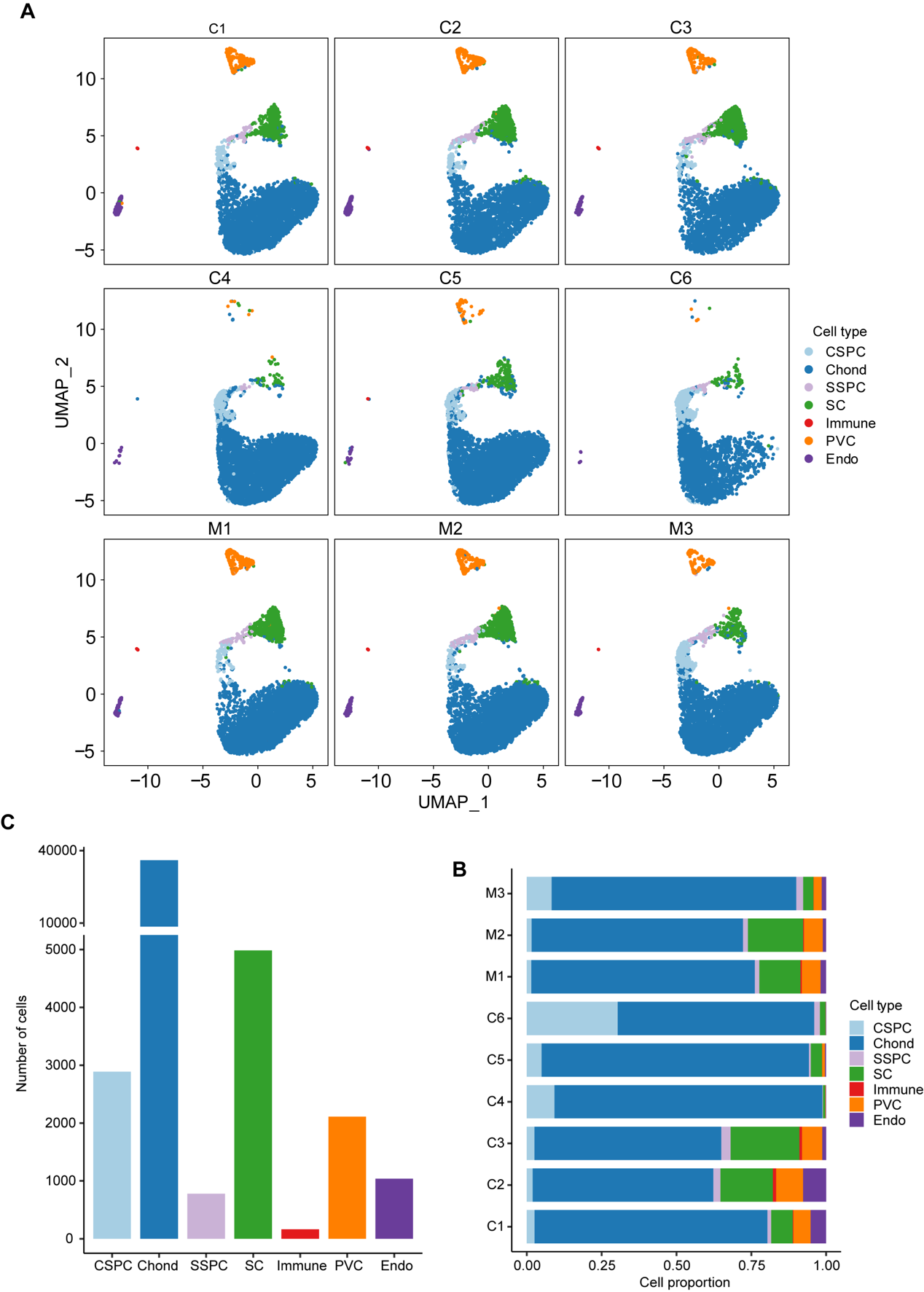


**Supplementary Figure 2. The distribution and proportion of cell types in human auricular cartilage.**

(A) UMAP projection showing the distribution of seven cell types in each sample. Among different samples, CSPC and Chond were the most abundant cell types in human auricular cartilage, and little or no representation cells by SSPC, SC, Immune, PVC, or Endo of C4 and C6 samples potentially due to incomplete or inconsistent dissection of the perichondrium layers.

(B) Bar plot of the number of each cell type in all samples.

(C) Bar plot of the percentage of each cell type in each sample.


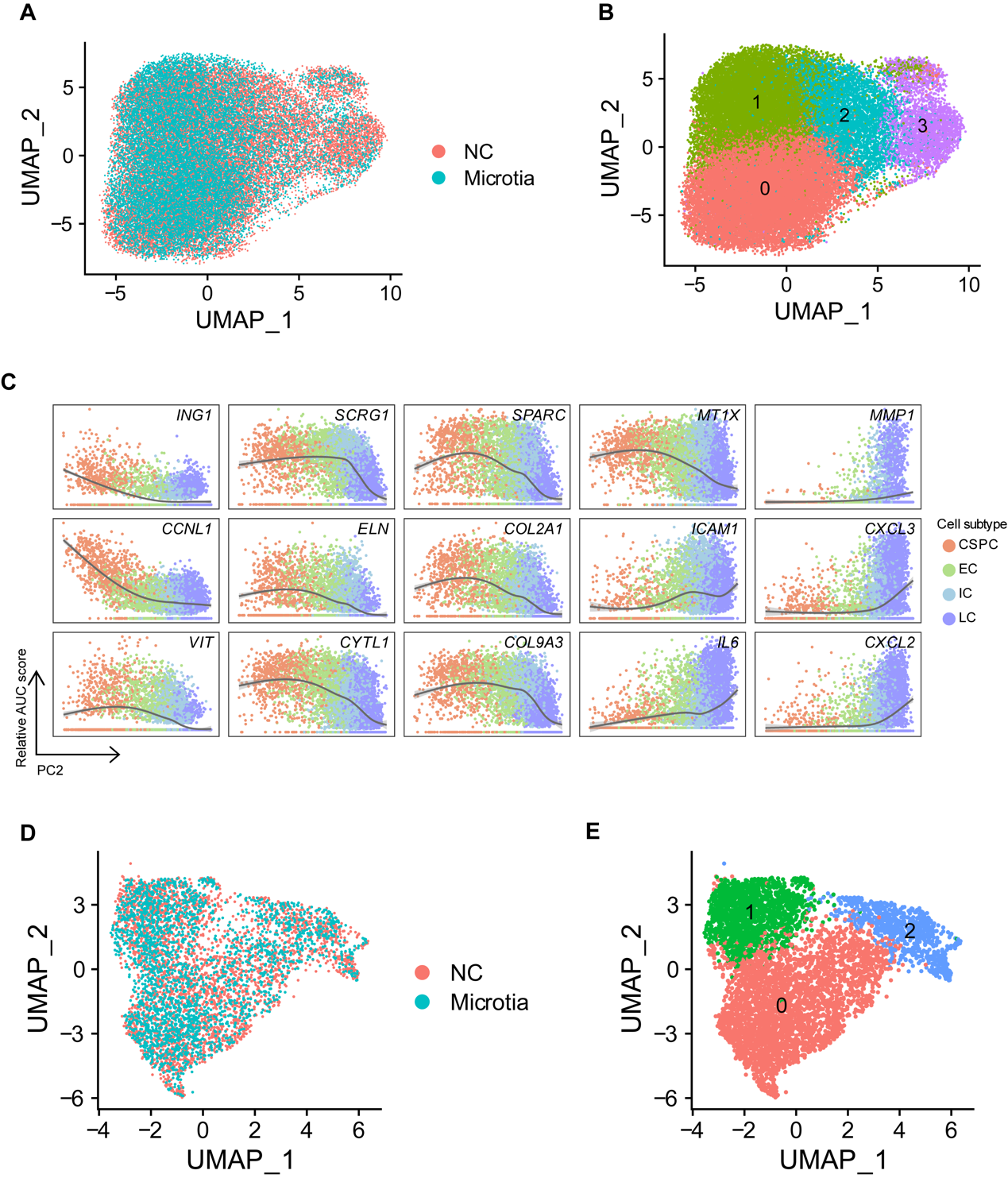


**Supplementary Figure 3. Characterization of the chondral and stromal lineages.**

(A-B) UMAP projections showing the distribution of the chondral lineage.

(C) Relative gene expression patterns of other representative genes essential for the chondral lineage along the PC2 dimension.

(D-E) UMAP projection showing the distribution of the stromal lineage.


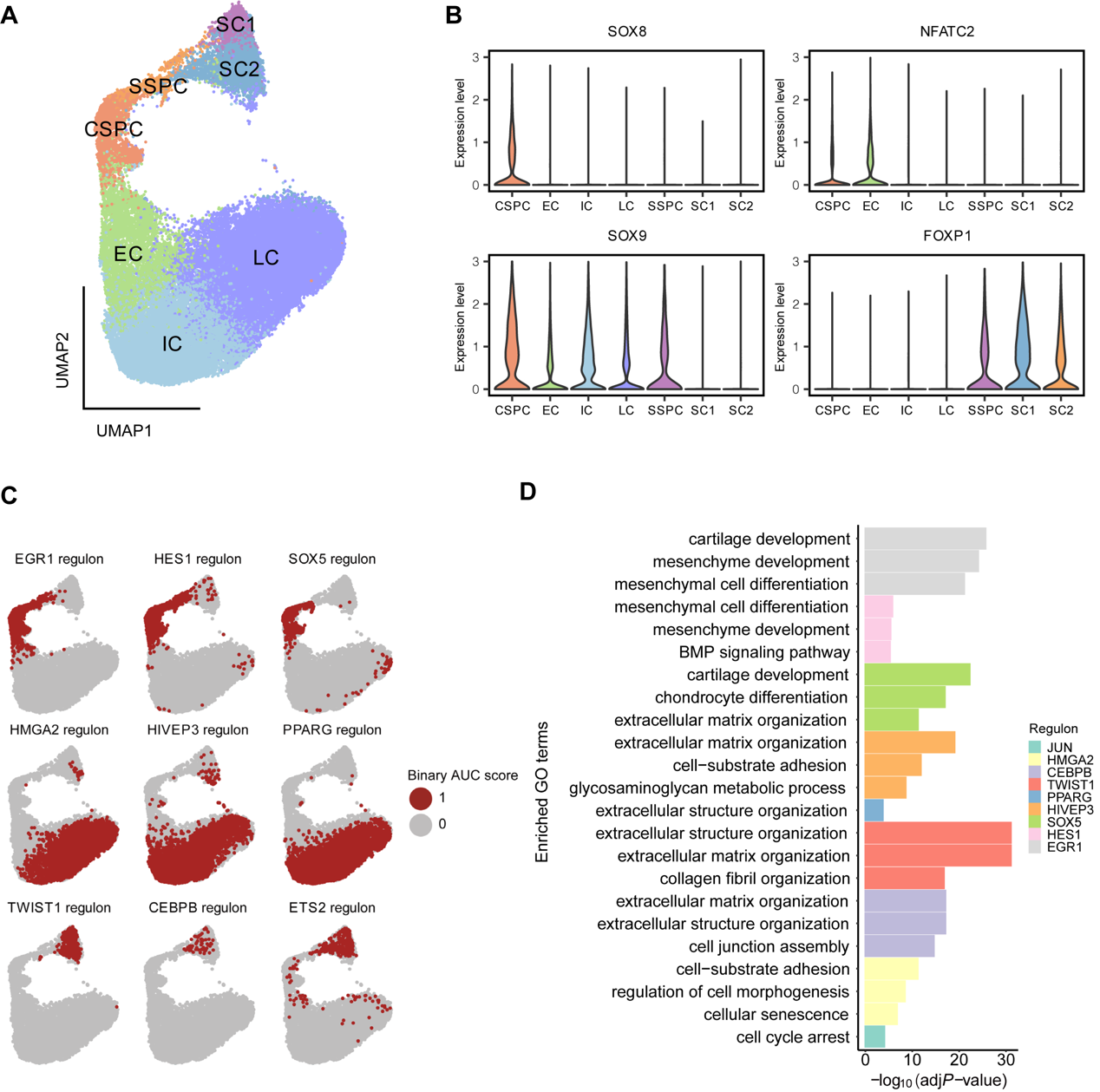


**Supplementary Figure 4. Cell subtype specific regulons in the chondral and stromal lineages**

(A) UMAP projection showing the distribution of the subtypes in the chondral and stromal lineages.

(B) Violin plots showing the expression levels of representative TF in each cell subtypes.

(C) UMAP plots of binary activities of other representative regulons.

(D) Bar plots displaying the representative GO terms enriched in above representative regulons.


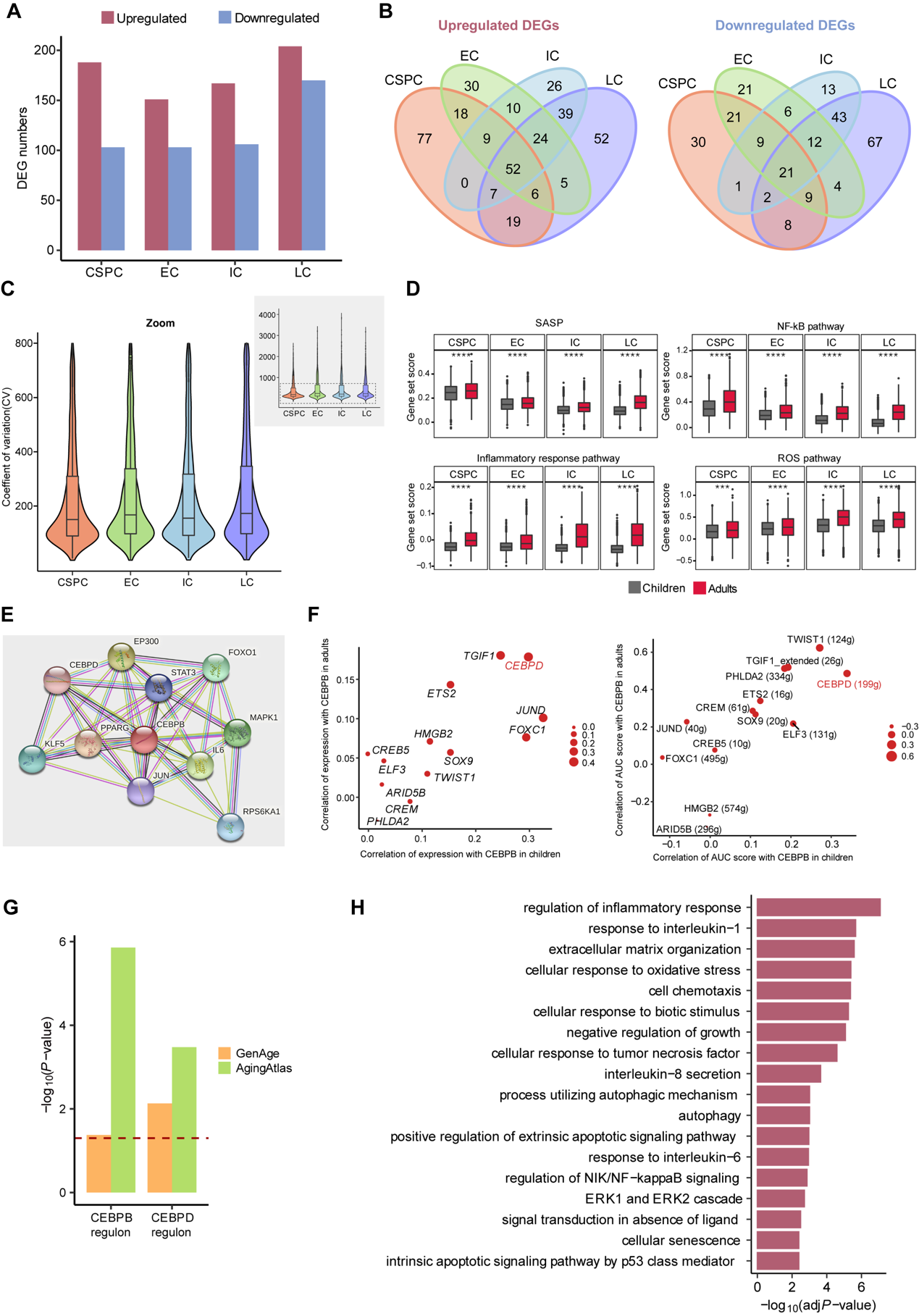


**Supplementary Figure 5**. **Age-associated changes in non-specific cell subtype manners in the chondral lineage between adults and children from NC group**

(A) Bar chart showing the number of DEGs identified in each subtype of the chondral lineage between adults and children.

(B) Venn plot of DEGs between adults and children in each subtype.

(C) CV analysis showing the age-associated transcriptional noise in the chondral lineage. Left shows the zoom-in view of the region highlighted by a dashed line on the top right.

(D) Boxplot plots showing gene set score of representative pathways in each subtype of adults and children. ***p < 0.001, ****p <0.0001.

(E) The protein-protein interaction (PPI) network of CEBPB and other TFs.

(F) The correlation of expression between CEBPB and other TFs and activity between CEBPB regulons and other regulons in the chondral lineage of adults and children. Dot size represents the correlation value.

(G) Bar plots showing the enrichment degree between upregulated DEGs of CEBPB and CEBPD regulons from children and adults with Aging Atlas and GenAge databases.

(H) Bar plots displaying the representative GO terms of the upregulated DEGs of CEBPB and CEBPD regulons.


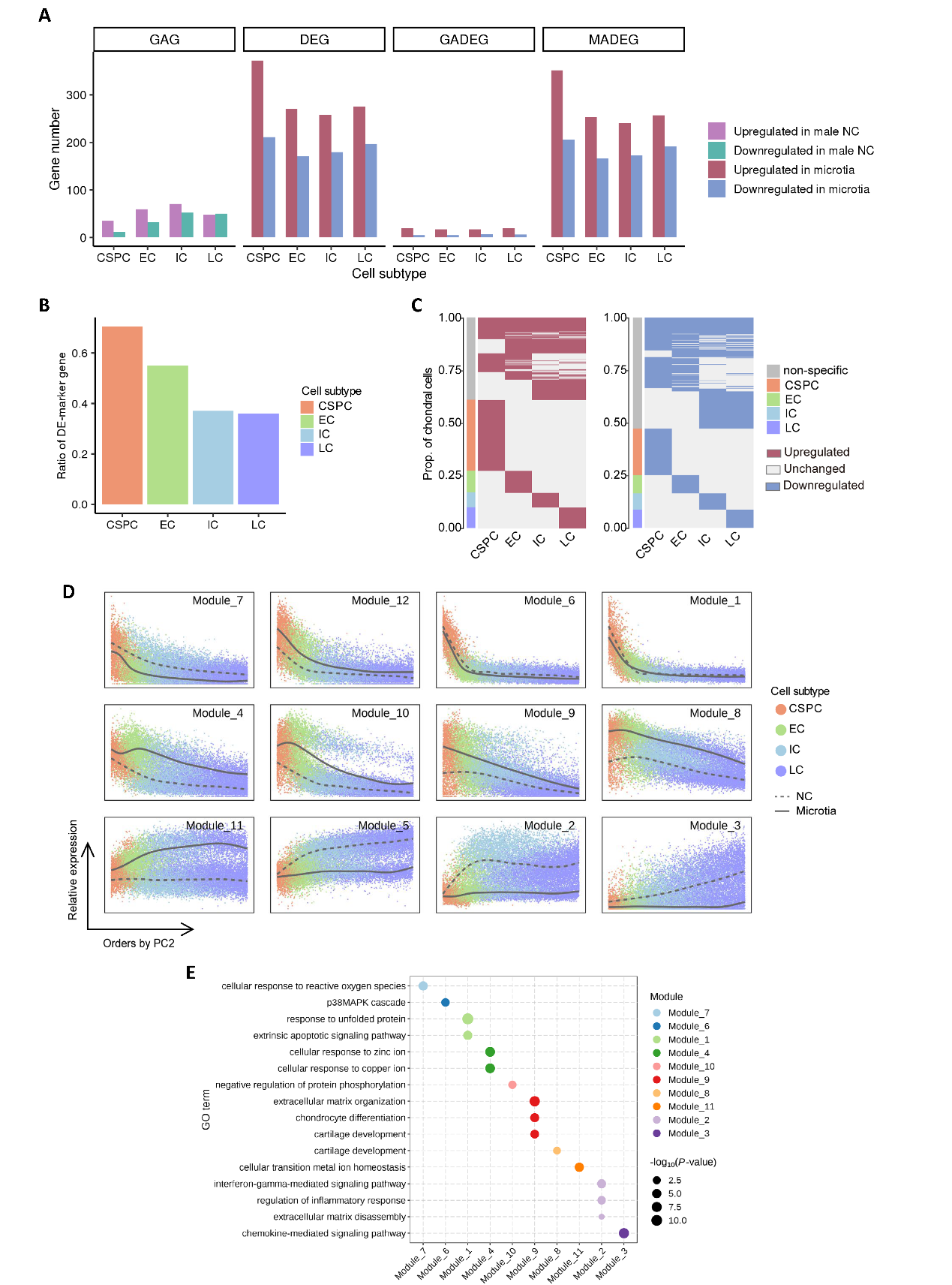


**Supplementary Figure 6. Analysis of the modules in the chondral lineage.**

(A) Bar charts showing the number of gender associated genes (GAGs), and differential expression genes (DEGs), gender associated DEGs (GADEGs) and microtia associated DEGs (MADEGs) between microtia and NC children in each subtype of the chondral lineage.

(B) Bar charts showing relative proportions of MADEGs between microtia and NC children in marker genes of each subtypes.

(C) Heatmaps showing the distribution of MADEGs between microtia and NC children in each subtype of the chondral lineage. Stacked bar charts on the left of the heatmaps show the relative proportions of MADEGs in each subtype; The gray blocks in the heatmaps denote MADEGs shared by at least two subtypes and the others are subtype specific MADEGs.

(D) Relative gene expression patterns of 12 modules along the PC2 orders dimension in microtia and NC children.

(E) Dots plots displaying the representative GO terms enriched in modules.


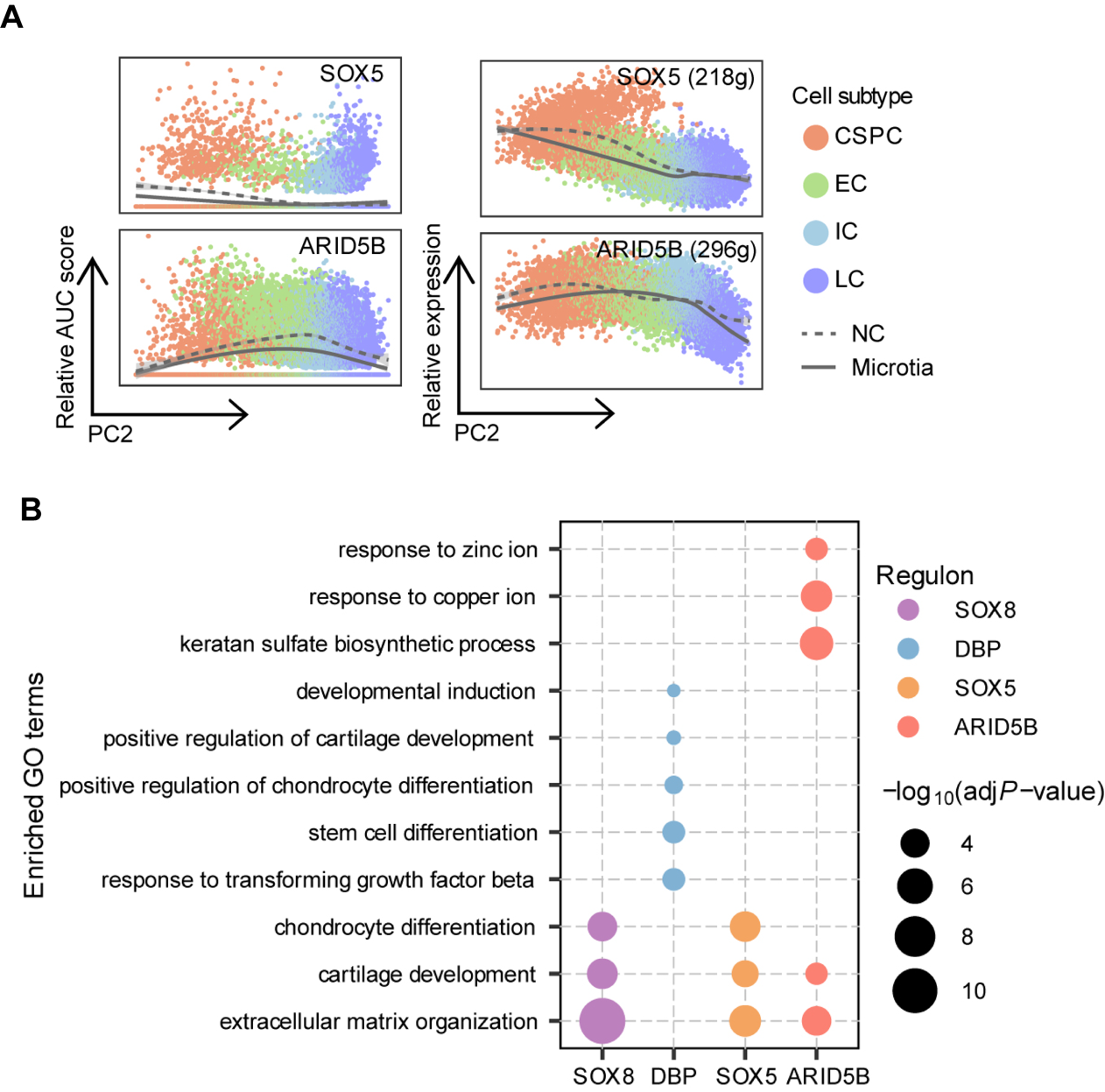


**Supplementary Figure 7. Downregulated regulons of CSPCs in microtia.**

(A) Relative gene expression of SOX5 and ARID5B, and AUC score of SOX5 and ARID5B regulons along the PC2 dimension of microtia and NC children.

(B) Dot plots displaying the representative GO terms enriched in downregulated regulons of microtia. MADEGs targeted by SOX8, SOX5, and ARID5B participated in regulation of ECM organization and cartilage development. DBP and its target MADEGs were related to stem cell differentiation and positive regulation of cartilage development.


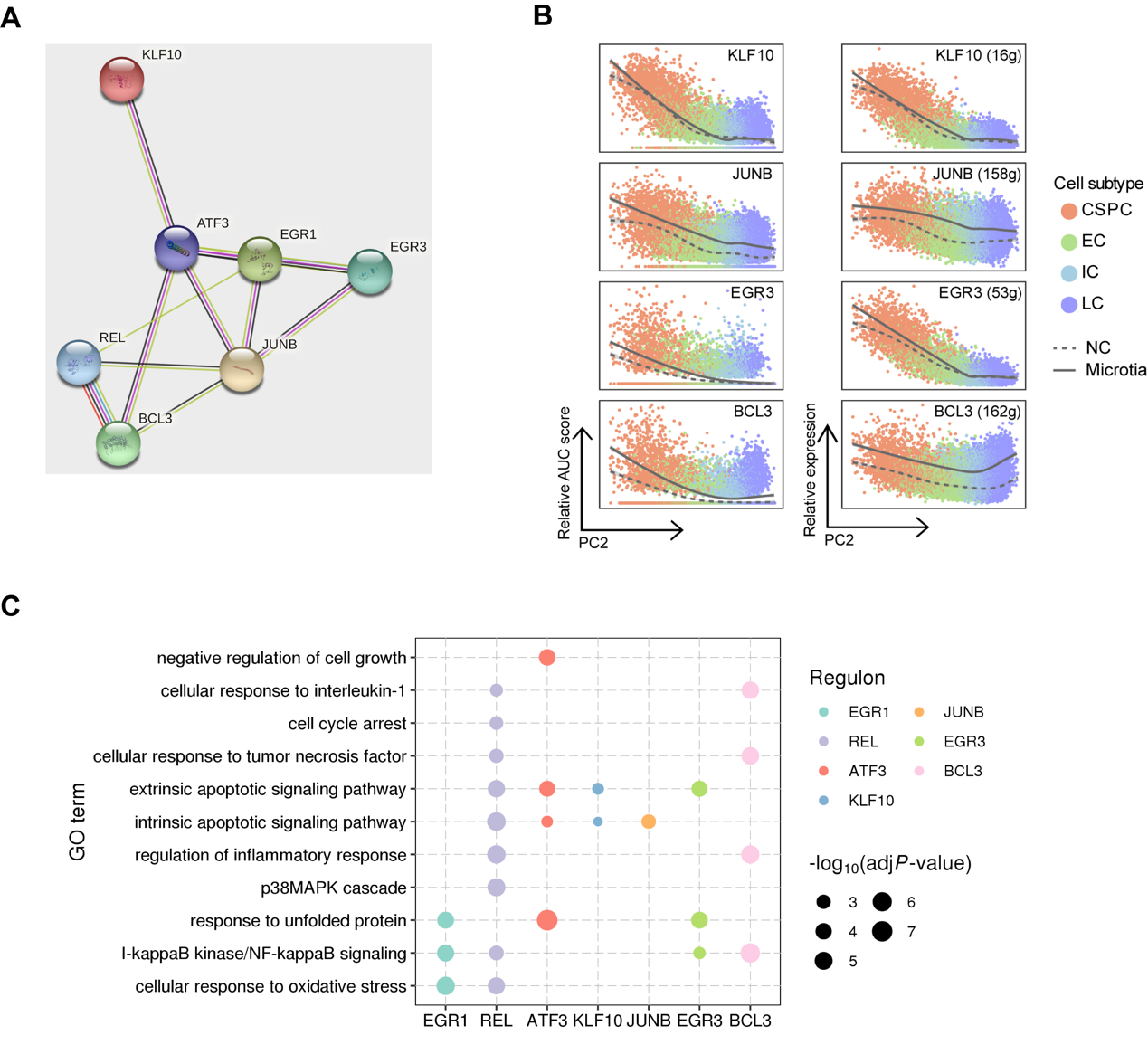


**Supplementary Figure 8. Upregulated regulons of CSPCs in microtia.**

(A) The PPI network of upregulated TFs in microtia.

(B) Relative gene expression of KLF10, JUNB, EGR3, BCL3, and AUC score of KLF10, JUNB, EGR3 and BCL3 regulons along the PC2 dimension of microtia and NC children.

(C) Dot plots displaying the representative GO terms enriched in upregulated regulons of microtia. GO analysis suggested that these TFs and their target MADEGs were mainly related to response to oxidative stress, I-kappaB kinase/NF-kappaB signaling pathway, apoptotic signaling pathway, and inflammatory response.


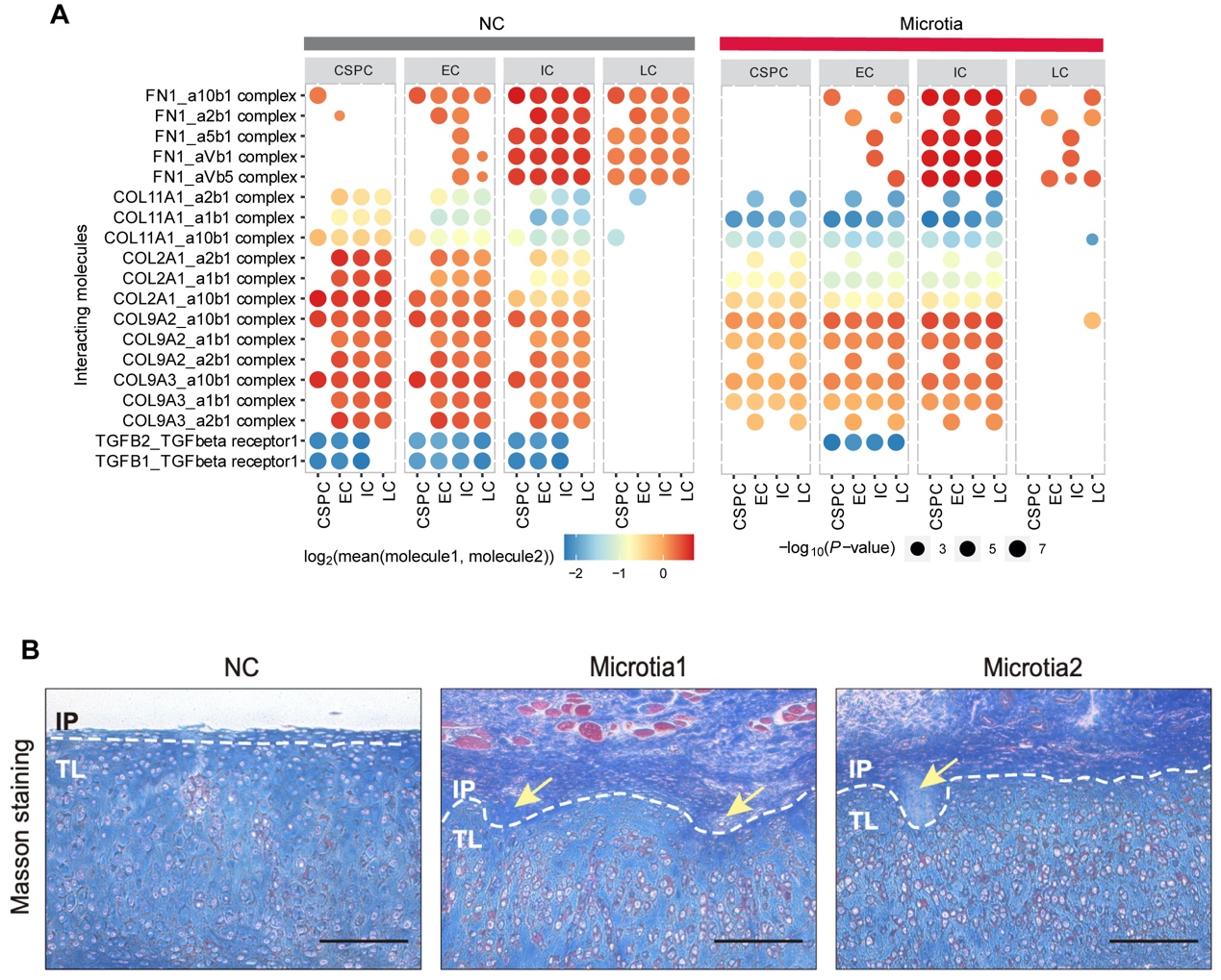


**Supplementary Figure 9**. **Ligand-receptor interactions in microtia.**

Interactions between integrins and collagens/fibronectins were generally weakened in microtia compared to that in NC children (A), contributing to the failure to form regular, multi-layer cartilage tissue structures (B).

(A) Dot plots showing selected ligand-receptor interactions of four subtypes in the chondral lineage between NC children and microtia. The ligand-receptor interactions are indicated at columns. The means of the average expression levels of two interacting molecules complex are indicated by color dots. For example, interactions between FN1 (secreted from LCs) and five isoforms of the integrin complex on the surface of four subtypes were either weakened or disappeared altogether in microtia. Similarly, the strength of interactions between collagens secreted from CSPCs and different integrin complexes were also weakened in microtia.

(B) Masson’s trichrome staining of NC (*N*=1) and microtia (*N*=2) auricular cartilage. Dashed lines denote the boundary between the inner perichondrium (IP) and the transition layer (TL); The yellow arrows show the disrupted structure. Scale bar, 200 μm.
